# Efficacy of Vitamin D Combined with Metformin and Clomiphene in the Treatment of Patients with Polycystic Ovary Syndrome Combined with Infertility

**Published:** 2019-10

**Authors:** Lili ZHUANG, Wei CUI, Jianxiang CONG, Yinghong ZHANG

**Affiliations:** 1. Reproductive Department, Yantai Yuhuangding Hospital, Yantai 264000, P.R. China; 2. Obstetrics Department, Yantai Yuhuangding Hospital, Yantai 264000, P.R. China

**Keywords:** Vitamin D, Metformin, Clomiphene, Polycystic ovary syndrome, Infertility

## Abstract

**Background:**

We aimed to explore the clinical efficacy of vitamin D combined with metformin and clomiphene in the treatment of patients with polycystic ovary syndrome combined with infertility.

**Methods:**

Overall, 396 cases of polycystic ovarian syndrome combined with infertility in Yantai Yuhuangding Hospital, Yantai, China were prospectively analyzed. Among them, 204 cases treated with vitamin D combined with metformin and clomiphene were set as the study group; 192 cases treated with only metformin and clomiphene were set as the control group. The ovarian volume and thickness of uterine wall before and after treatment were recorded. Levels of fasting insulin (FINS), luteinizing hormone (LH), testosterone (T), follicle-stimulating hormone (FSH), and estradiol (E2) before and after treatment were recorded.

**Results:**

There was no difference in body weight, BMI, ovarian volume, thickness of ovarian wall, FINS, LH, T, FSH and E2 between the study group and the control group; there was no significant difference in FSH, E2 and the thickness of uterine wall between the two groups. After treatment, the BMI, FINS, LH, and T in the study group were significantly lower than those in the control group (*P*<0.05); the incidence rates of oligomenorrhea, facial acne and hairy symptoms in the study group were significantly lower than those in the control group (*P*<0.05); after treatment, the ovulation rate and pregnancy rate in 36 cycles in the observation group were significantly higher than those of the control group (*P*<0.05).

**Conclusion:**

Our challenge could significantly improve clinical symptoms and endocrine conditions, and greatly enhance the ovulation rate and pregnancy rate.

## Introduction

Polycystic Ovary Syndrome (PCOS) is the most common endocrine disease in women, and its morbidity is about 7% of women of childbearing age, which is the main reason for anovulatory infertility (1, 2). About 74% of the 1079 patients with PCOS have infertility and have a high rate of morbidity (3). In recent years, due to the declining fertility rate in developed countries in Europe and America, the problem of fertility has become increasingly severe. The problem of infertility caused by physical diseases has also received more and more attention from the medical community (4), and PCOS infertility is one of them.

At present, the pathogenesis of PCOS is still unclear. Because the age of onset is from puberty to 40, considering fertility demand factors and treatment effects of patients, drug treatment is still the first and most effective way to treat PCOS infertility (5). Clomiphene (CC), as a first-line treatment for PCOS, has few side effects, is inexpensive and easy to buy, and is widely used, but 15% to 40% of patients are resistant to this drug (6). Metformin (MF) is currently the most widely used insulin sensitizer in the world and is a very adaptive drug for the treatment of PCOS (7). MF combined with CC could be effectively applied to patients with CC resistance (8). Vitamin D (VD), such an oral, relatively safe, and inexpensive vitamin, could help to treat common ovulation dysfunction in PCOS by promoting follicular development, improve menstruation, etc., and it may be used in infertility in all women of childbearing age (9, 10). Adequate VD levels (≥30 ng/ml) should be required in women undergoing external ejaculation, and the lack of VD may aggravate the symptoms of PCOS (11,12).

In this study, after examining the VD level of patients with PCOS, VD was added to the combination of MF and CC treatment, to see if the efficacy on PCOS and the therapeutic effect on infertility could be effectively improved. Therefore, this study prospectively analyzed the efficacy of VD combined with MF and CC in the treatment of PCOS infertility, in order to provide references for drug treatment of PCOS.

## Materials and Methods

### Objects of Study

Overall, 396 patients with PCOS combined with infertility admitted to Yantai Yuhuangding Hospital, Yantai, China were retrospectively analyzed, aged from 20 to 40 yr, and they were divided into two groups according to different treatment methods, including 204 patients receiving VD combined with MF and CC treatment (study group) and 192 patients receiving MF and CC treatment (control group).

The inclusion criteria were as follows: All patients were in accordance with PCOS diagnostic criteria, and all patients were treated with drugs. The clinical data of patients were complete. The exclusion criteria were as follows: Patients with kidney failure, kidney stones and medical history including digestive system diseases that are in the active stage, patients with adrenocortical hyper-function, abnormal liver function, kidney function and cardiopulmonary function, pregnant women or women in lactation period, and patients with mental diseases or abnormal judgment of brains.

This study was approved by the Medical Ethics Committee of Yantai Yuhuangding Hospital, and all patients and their families were informed by letters or telephones, and signed informed consent forms.

### Drug Therapy

The control group was administered with MF (Tianjin Zhongxin Pharmaceutical Group Co., Ltd. Xinxin Pharmaceutical Factory, SFDA approval number: H12020587) combined with CC (Guangzhou Kanghe Pharmaceutical Co., Ltd., SFDA approval number: H44021970): 100 mg of CC once a time and once a day and 500 mg of MF once a time and three times a day on the 5^th^ day of the menstrual cycle were taken orally; the medication was stopped after continuous medication for five days, and continued on the 5^th^ day of the next menstrual cycle, lasting three consecutive menstrual cycles. The research group was administered with VD (Qingdao Shuangjing Pharmaceutical Co., Ltd., SFDA approval number: 20113033) combined with MF and CC: On the basis of the control group, 3000 IU of VD once a time and once a day were added, lasting three consecutive menstrual cycles.

### Observation Indicators

General data of patients, including age, body mass index (BMI), menstrual conditions, etc., were collected; VD levels before and after treatment in both groups were compared; levels of fasting serum lisulin (FINS) and luteinizing hormone (LH), testosterone (T), follicle stimulating hormone (FSH) and estradiol (E2) of patients before and after treatment were observed. The incidence rates of oligomenorrhea, facial acne, hairy symptoms and the ovulation rate and pregnancy rate of 36 cycles of patients in the two groups were observed.

### Statistical Methods

SPSS19.0 (Asia Analytics Formerly SPSS, China) was used. The measurement data were expressed as (n(%)), and the ratio between the two groups was compared by the *χ*^2^ test. The count data were expressed as mean±standard deviation (mean±sd). The comparison between the two groups was performed by independent sample t test. When *P* was less than 0.05, there was statistically significant difference.

## Results

### General Data

There were 192 patients in the control group, aged (25.64±4.78), and 204 patients in the study group, aged (26.33±4.05). There was no significant difference of age between the two groups. The comparison of other data of patients in the two groups such as menstrual cycle and duration, duration of infertility also showed no statistical significance (Table 1).

**Table 1:** General Dataof study and control group

***Variable***	***Control group (n=192)***	***Study group (n=204)***	****χ*^2^/*t**	**P *value***
Age (yr)	25.64±4.78	26.33±4.05	0.121	1.553
Menstrual cycle (d)	31.21±17.56	30.55±16.82	0.382	0.703
Menstrual duration (d)	5.21±4.53	5.46±5.36	0.500	0.618
History of reproduction (n(%))			0.107	0.743
Yes	37 (19.27)	42 (20.59)		
No	155 (80.73)	162 (79.41)		
Duration of infertility (y)	3.52±2.56	3.87±2.44	1.393	0.164
Enlargement of ovary (n(%))			0.436	0.509
Yes	100 (52.08)	113 (55.39)		
No	92 (47.92)	91 (44.61)		
Smoking history (n(%))			0.629	0.802
Yes	58 (30.21)	64 (31.37)		
No	134 (69.79)	140 (68.63)		
Alcohol history (n(%))			0.162	0.688
Yes	98 (51.04)	100 (49.02)		
No	94 (48.96)	104 (50.98)		

### Analysis of VD levels

**Fig. 1:**
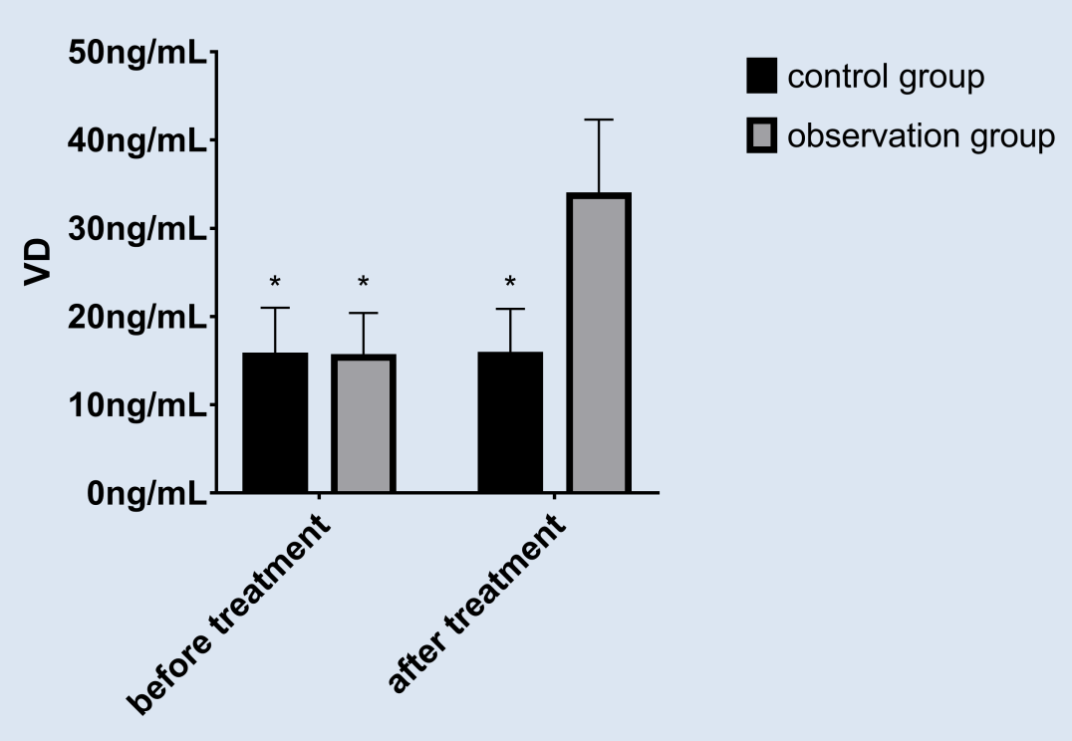
VD level analysis.There was no significant difference in VD levels between the two groups before treatment; there was no significant difference before and after treatment in the control group; the VD level in the study group was significantly higher than that in the control group after treatment, and the difference was statistically significant; the VD level in the study group was significantly higher than that before treatment, the difference was statistically significant. * indicated *P* <0.05

There was no significant difference in VD levels between the two groups before treatment. After treatment, the VD level of the study group was significantly higher than that of the control group (*P*<0.05). After treatment, the VD level of the study group was significantly higher than that before treatment (*P*<0.05). There was no significant difference in the control group before and after treatment (*P*>0.05) (Fig. 1).

### Analysis of Hormone Indicators

The FINS, LH and T of the two groups after treatment were lower than those before treatment, and the study group was more significant (P<0.05). But there was no significant difference between the FSH and E2 before and after treatment. After treatment, the FINS, LH and T of the study group were significantly lower than those of the control group, and the difference was statistically significant (*P*<0.05) (Table 2).

**Table 2:** Analysis of hormone indicators

***Variable***	***Control group (n=192)***	***Study group (n=204)***	**t**	**P *value***
FINS (pmol/L)				
Before treatment	98.26±46.41	102.26±40.32	0.917	0.360
After treatment	78.20±36.25^*^	53.62±35.24^*^	6.841	<0.001
LH (IU/L)				
Before treatment	13.79±8.64	14.49±6.21	0.930	0.353
After treatment	11.56±7.62^*^	6.35±6.68^*^	7.246	<0.001
T(nmol/L)				
Before treatment	1.79±0.87	1.87±0.68	1.023	0.307
After treatment	1.15±1.04^*^	0.45±0.51^*^	8.580	<0.001
FSH (IU/L)				
Before treatment	7.48±1.67	7.26±2.03	1.174	0.241
After treatment	7.56±1.75	7.54±1.79	0.112	0.910
E2 (pmol/L)				
Before treatment	178.32±45.68	180.61±48.26	0.484	0.628
After treatment	176.52±42.69	180.52±46.25	0.893	0.373

Note:

* indicated that P was less than 0.05 before treatment in the same group

### Analysis of Related Signs

The incidence rates of oligomenorrhea, facial acne and hairy symptoms after treatment in the study group were significantly lower than those in the control group, and the difference was statistically significant (*P*<0.05). After treatment, the ovulation rate and pregnancy rate of the study group were significantly higher than the control group in 36 cycles (*P*<0.05) (Table 3).

**Table 3:** Analysis of related signs

***Variable***	***Control group (n=192)***	***Study group (n=204)***	***χ^2^***	**P *value***
Oligomenorrhea(n(%))	43 (22.40)	18 (8.82)	13.982	<0.001
Facial acne(n(%))	35 (18.23)	21 (10.92)	5.129	0.024
Hairy symptoms (n(%))	37 (19.27)	19 (9.31)	8.077	0.005
Ovulation rate (n(%))	106 (55.21)	188 (92.16)	70.611	<0.001
Pregnancy rate (n(%))	89 (46.35)	136 (66.67)	16.633	<0.001

### Analysis of Ovarian and Uterine Changes

The left ovarian volume and right ovarian volume after treatment were smaller than those before treatment (*P*<0.05), and the thickness of uterine wall had no significant difference before treatment. The left ovarian volume and right ovarian volume after treatment of the study group were smaller than those of the control group, and there was no significant difference in the thickness of the uterine wall (*P*<0.05) (Table 4).

**Table 4:** Analysis of ovarian and uterine changes

***Variable***	***Control group (n=192)***	***Study group (n=204)***	**t**	**P *value***
Left ovarian volume (ml)				
Before treatment	8.67±2.38	8.72±2.66	0.197	0.844
After treatment	7.23±1.68*	5.54±1.21*	11.537	<0.001
Right ovarian volume (ml)				
Before treatment	8.58±2.64	8.45±2.36	0.517	0.605
After treatment	7.15±1.88*	5.87±1.43*	7.653	<0.001
Thickness of uterine wall (mm)				
Before treatment	6.89re tr	6.67re tr	0.741	0.459
After treatment	6.75r tre	6.62r tre	0.437	0.662

Note:

* indicated that *P* was less than 0.05 before treatment in the same group

### Analysis of Body Mass index

After treatment, the body weight and BMI of the two groups were lower than those before treatment, and the study group had a more significant decrease (*P*<0.05) (Table 5).

**Table 5:** Analysis of body mass index

***Variable***	***Control group (n=192)***	***Study group (n=204)***	**t**	**P *value***
Body weight (kg)				
Before treatment	62.26±12.62	61.25±13.99	0.753	0.452
After treatment	59.24±10.52^*^	56.46±8.58^*^	2.889	0.004
BMI (kg/m2)				
Before treatment	27.28±3.56	27.53±4.13	0.643	0.520
After treatment	26.26±2.57^*^	25.72±1.86^*^	2.406	0.017

Note:

* indicated that P was less than 0.05 before treatment in the same group

### Analysis of Adverse Reactions

There were no significant differences in adverse reactions of nausea, vomiting and diarrhea of patients between the two groups during the treatment. There were no significant differences in total adverse reactions. No serious adverse reactions of patients occurred in the two groups (Table 6).

**Table 6:** Analysis of adverse reactions

***Variable***	***Control group (n=192)***	***Study group (n=204)***	***χ^2^***	**P *value***
Nausea	10 (5.21)	12 (5.88)	0.086	0.770
Vomiting	11 (5.73)	10 (4.90)	0.135	0.714
Diarrhea	8 (4.17)	12 (5.88)	0.607	0.436
Total adverse reactions	29 (15.10)	34 (16.67)	0.181	0.671

## Discussion

As the most common endocrine disease in women of childbearing age, PCOS can cause irregular menstruation, infertility and elevated androgen levels, leading to hirsutism and acne, which seriously affect women's quality of life and psychological conditions (higher risk of depression).

Their fertility needs have also been severely hampered, and PCOS infertility is the most important factor in female infertility (13–15).

At present, the most commonly used method for the treatment of PCOS is still drug therapy. As the first-line drug CC and MF, it has been reported that the ovulation rate of MF combined with CC drug (83.3%) is significantly higher than that of MF alone (56.2%) and CC alone (62.5%), but the pregnancy rate (50%) was lower than that of MF alone (54.2%), and effects were not fully confirmed by the clinic (16). Vitamin D may play an important role in increasing the ovulation rate and pregnancy rate in women with PCOS (17). Supplementation with VD had as a positive effect on weight loss, follicular maturation, menstrual regularity, and improvement of hyperandrogenism in PCOS infertile women (18, 19). These findings provide ideas for the treatment of PCOS infertility in this study, that is, on the basis of MF and CC medication, whether or not the addition of VD can have better therapeutic effect. Therefore, this study compared clinical effects of VD combined with MF and CC versus MF and CC on the treatment of PCOS infertility, in order to provide references for clinical drug treatment of PCOS infertility.

The VD level of PCOS patients is significantly lower than that of healthy people, and low VD may induce PCOS, and low VD is associated with menstrual disorders (19), suggesting a causal relationship between low VD level and PCOS onset. VD-deficient PCOS females supplemented with VD could significantly reduce the bioavailability of transforming growth factor-β1 (TGF-β1), so as to effectively improve the levels of BMI, T, FSH, LH, etc., which may be one of its mechanisms of action (20). Vitamin D supplementation could help pregnant women with PCOS and insulin resistance restore levels of vitamin D in serum to normal levels, thereby improving embryo quality and significantly increasing clinical pregnancy rates (21). These two studies showed that VD treatment was effective for PCOS patients. In addition, one study showed that vitamin D deficiency reduced the pregnancy rate in women undergoing a single blastocyst transplant (22), and another study indicated that taking VD six weeks prior to intracytoplasmic sperm injection could effectively improve endometrial quality and clinical pregnancy rate (23).

From these two studies, we suspected that in the absence of VD, the impact on women was not only limited to PCOS infertility, but also women may be infertile under different symptoms or normal conditions. One study indicated that supplementation with 50,000 IU of vitamin D every other week for 8 weeks had beneficial effects on insulin metabolism and blood lipids in infertile women with polycystic ovary syndrome who were candidates for in vitro fertilization, but these benefits may not be apparent when vitamin D levels are adequate (24). We considered that patients with PCOS infertility with adequate VD levels may not be very effective. Interestingly, one study pointed out that low VD level in men would also affect their fertility, indicating that VD level had a huge impact on human fertility (25). In future clinical treatment, the test result of VD level may be one of the important reference standards.

Of course, because it is a prospective study, this study also had some shortcomings. Inevitably, there are some selection bias, although we have narrowed the range of age which only included patients aged from 20 to 40 years. The number of our sample did not reach the ideal. In addition, our study did not collect the physical condition and re-pregnancy within five years after treatment, and we hope to improve our research in future randomized controlled trials of large samples. In terms of FSH and E2 indicators, there was no significant difference between the study group and the control group. This study did not explore its specific reasons, which is one of our future research direction.

## Conclusion

The clinical efficacy of VD combined with MF and CC in the treatment of infertility patients with polycystic ovary syndrome is better than that of MF and CC, which has reference significance for the clinical treatment of PCOS infertility.

## Ethical considerations

Ethical issues (Including plagiarism, informed consent, misconduct, data fabrication and/or falsification, double publication and/or submission, redundancy, etc.) have been completely observed by the authors.
